# Development of a Low-Cost Sensor System for Accurate Soil Assessment and Biological Activity Profiling

**DOI:** 10.3390/mi15111293

**Published:** 2024-10-24

**Authors:** Antonio Ruiz-Gonzalez, Harriet Kempson, Jim Haseloff

**Affiliations:** Department of Plant Sciences, University of Cambridge, Downing St., Cambridge CB2 3EA, UK; a.gonzalez.16@ucl.ac.uk (A.R.-G.); harriet.kempson@bristol.ac.uk (H.K.)

**Keywords:** microsensor, quorum sensor, e-nose, artificial neural network

## Abstract

The development of low-cost tools for rapid soil assessment has become a crucial field due to the increasing demands in food production and carbon storage. However, current methods for soil evaluation are costly and cannot provide enough information about the quality of samples. This work reports for the first time a low-cost 3D printed device that can be used for soil classification as well as the study of biological activity. The system incorporated multiple physical and gas sensors for the characterisation of sample types and profiling of soil volatilome. Sensing data were obtained from 31 variables, including 18 individual light wavelengths that could be used to determine seed germination rates of tomato plants. A machine learning algorithm was trained using the data obtained by characterising 75 different soil samples. The algorithm could predict seed germination rates with high accuracy (RSMLE = 0.01, and R^2^ = 0.99), enabling an objective and non-invasive study of the impact of multiple environmental parameters in soil quality. To allow for a more complete profiling of soil biological activity, molecular imprinted-based fine particles were designed to quantify tryptophol, a quorum-sensing signalling molecule commonly used by fungal populations. This device could quantify the concentration of tryptophol down to 10 nM, offering the possibility of studying the interactions between fungi and bacterial populations. The final device could monitor the growth of microbial populations in soil, and offering an accurate assessment of quality at a low cost, impacting germination rates by incorporating hybrid data from the microsensors.

## 1. Introduction

The optimisation of land management and improved soil use is key in tackling the UN development goals [1]. In particular, the development of technologies to address current challenges in crop yields is crucial to ensure the 70% increase in agricultural production needed by 2050 [2,3]. However, the prevalence of intensive agricultural methods and the continued use of malpractices such as tilling [4] or fertiliser overuse [5] have progressively reduced soil quality, leading to millions of crop losses. In the U.K., it is estimated that the effects of degradation due to contamination, carbon loss, or erosion, among others, causes up to GBP 1.2 billion of losses every year [6]. However, current techniques for soil assessment, which can be used to optimise farming techniques, are expensive, and they lack accuracy.

Hyperspectral imaging techniques represent one of the most widespread methods used so far for the non-invasive monitoring of soil. These methods rely on the measurement of the light intensity reflected by soil. This light intensity at specific wavelengths can be correlated with the presence of multiple components, including nitrogen [7], phosphorous [8], and potassium [9] in soil, which are crucial for plant development. However, absorbed light is also influenced by soil type and differences in texture, mineral content, moisture, and presence of organic carbon, among others [10,11], which limits the accuracy of these methods. Moreover, the assessment of soil health is complex, as it is also determined by multiple parameters, such as composition and soil microbiome [12]. Jia et al. [13] developed a hyperspectral imaging analysis method that could be used for the classification of soil samples and determination of total nitrogen. This imaging technique involves the measurement of near-infrared wavelengths (874–1734 nm). The determination of these parameters could additionally be used for the early detection of environmental hazards such as fertiliser overuse. However, this method requires the use of bulky and expensive equipment, which generate a large volume of data. As such, the results analysis become challenging, and the information provided by these sensors cannot be used for the study of biological activity in soil, which is crucial for plant growth.

Within recent years, new low-cost and portable technologies for soil characterisation have been developed to tackle these challenges. In most cases, these sensors are used for the determination of soil moisture, which is a crucial parameter in crop growth [14,15], using electrical parameters. Reflectometry-based devices, either in the form of time-domain reflectometry [16] or frequency-domain reflectometry [17], represent the most common technologies available commercially for soil assessment. However, despite the easy data processing and lower operational costs, the obtained measurements are also influenced by soil properties such as composition, textures, or even the presence of air gaps [18]. In addition, in most cases, the application of these devices is limited to the determination of soil moisture and cannot be used for the study of other parameters of interest, such as the activity of microbial populations. An indirect assessment of microbial activity in soil has recently been made possible through the measurement of open circuit potential of a carbon-based electrode [19]. By recording the activity of bacterial biofilms, this device could be used for the study of soil health. However, to allow an accurate diagnosis of soil status and study the impact different environmental conditions on soil quality, additional information from soil biological activity is required. Other approaches in this field include the use of biopolymers such as poly(3-hydroxybutyrate-co-3-hydroxyvalerate) [20]. This biodegradable conductive material could determine microbial decomposition through the measurement of decomposition over time. However, these sensors cannot be reused, increasing operational costs over time.

While physical properties of soil, such as texture, bulk density, and electric conductivity, are essential for plant growth, biological indicators offer a dynamic view of the soil ecosystem. Microbes play a key role in breaking down organic matter into simpler compounds that plants can absorb [21]. Within recent years, their role in the communication with plant roots [22] and their control over seed germination are being unveiled. This microbiome has been observed to enhance nutrient availability and promote germination through the release of signalling molecules [23]. Soil microorganisms generate a variety of organic volatiles as a result, which can be characterised and correlated with the quality. This pattern changes in the presence of contamination [24], environmental conditions [25], or even as a result of the presence of diseases [26].

A promising alternative for the minimally invasive and low-cost diagnosis of soil health is the measurement of soil volatiles, which can be achieved through spectral methods such as mid-infrared spectroscopy [27]. Zhu et al. developed an artificial intelligence-enhanced mid-infrared gas spectroscopy method for the measurement of isopropyl alcohol, a viral biomarker. Using this method, a 99.08% accuracy was achieved. Within recent years, electronic noses have also been developed. In this case, multiple gas sensors are incorporated to determine patterns in volatilome, which are released as a response to multiple environmental conditions. These patterns can be used in the early detection of abiotic stress of plants [28] and even the presence of specific pesticides in soil [29].

Sensors are, in most cases, based on a heated metal-oxide device that changes its electrical resistance due to the exposure to different volatiles, allowing the measurement of volatiles [30]. Volatiles released by soil are heavily influenced by the activity of degradative bacteria and fungi [31] as well as plant growth [32] and stress factors such as drought [28]. As such, the determination of soil volatiles has become instrumental for the study of soil health. Following this concept, De Cesare et al. [33] designed an electronic nose that could be used for the study of microbial activity in soil. This new method could be used for the study of soil microbial activity, which is typically measured indirectly through the activity of multiple enzymes, such as the fluorescein diacetate hydrolase [34]. By allowing the detection of multiple volatiles, the metabolic fingerprint of certain bacteria such as *P. fluorescens* could be identified, and the growth in the populations could be evaluated. A similar approach was adapted by Bieganowski et al. [35], who developed an electronic nose that could be used for the study of soil contamination due to petroleum fuel. This device incorporates a classification algorithm and an artificial neural network that allow the identification of contamination source and study the status of the samples. Thus, the use of electronic noses represents a promising approach to tackle the current need for a low-cost, easy-to-use device for the study of soil health. However, despite the capability of these systems of determining volatile fingerprints in soil, they cannot be used for the complete evaluation of soil health, which requires a simultaneous assessment of soil composition and microbial activities. This multiplexed evaluation of soil can only be achieved by combining chemical sensors for produced gas volatiles and physical sensors and could be used to boost crop production by allowing decisions to be made about land management initiatives.

Current developments in soil health assessment require the use of expensive equipment, and they cannot provide a complete assessment of soil. This complete assessment includes the determination of abiotic factors such as electrical impedance and spectral emissions as well as biotic samples to evaluate microbiome composition. The present work reports for the first time a soil-testing device designed for the simultaneous classification of soil samples and study biological activity in a low-cost setting. This device combined gas and physical sensors (i.e., spectrometer and impedance) to offer a complete assessment of soil characteristics and biological activities.

The aim of this research is to develop and validate this novel, low-cost soil-testing device capable of providing a comprehensive assessment of soil health by simultaneously evaluating abiotic factors and biotic components and to demonstrate the feasibility of incorporating this device for soil fertility evaluation. Different machine learning algorithms were tested to predict soil quality and its impact on plant health, with a specific focus on improving the accuracy and accessibility of soil health diagnostics. To allow an accurate quantification of fungal populations, a sensing device containing molecularly imprinted silica microparticles was also developed. This sensor could quantify tryptophol, a quorum-sensing molecule for certain fungal colonies. In addition, the measurement of produced volatiles and open circuit potential could be used for the determination of general microbial activity. These sensors could be used to study the impact of bacterial and fungal growth on seed germination rates.

## 2. Materials and Methods

### 2.1. Materials

All reagents were purchased from Sigma Aldrich (St. Louis, MO, USA), unless otherwise specified: β-cyclodextrin (>97%) and p-toluenesulphonyl chloride (>99%), NaOH (>98%), ammonium chloride (99.998%), sodium alginate, Indoxyl sulphate (>97%), tryptophol, and N-(3-oxododecanoyl)-L-Homoserine Lactone (>98%). Poly (dimethylsiloxane) (PDMS) was purchased from Dow Inc. (Midland, MI, USA).

The heating element, methacrylate sheet NTC-type sensor, Capacitive moisture sensor, CO_2_ sensor (SCD30), BME680, 10 kOhm resistor, and WISKA Combi Junction Box (IP66) were purchased from RS components (Corby, UK). The Wio Terminal, relay, and MCP4725 (Analog-to-digital converter) were purchased from Seeed Studio (Shenzhen, China). Impedance analyser ad5933 AS7265x triad was purchased from Sparkfun (Niwot, CO, USA). An LM324 Operational amplifier was purchased from Texas Instruments (Dallas, TX, USA). An air pump was purchased from Pssopp (UK). Screen printed electrodes were purchased from Saida Technology (Suzhou, China). Compost samples were purchased from Mokuzai (Kingston Upon Thames, UK) and Miracle-Gro (Marysville, OH, USA). Finally, tomato seeds (Gardeners delight variety) were purchased from Thompson (Thompson, MI, USA) and Morgan (Morgan, Malvern, UK). Mycorrhizal fungi were purchased from vitax (Coalville, UK). Analog gas sensors (MQ2, MQ3, MQ4, MQ7, and MQ131) were purchased from Cool Components (London, UK).

### 2.2. Design of Soil Chamber

**Figure A1:**
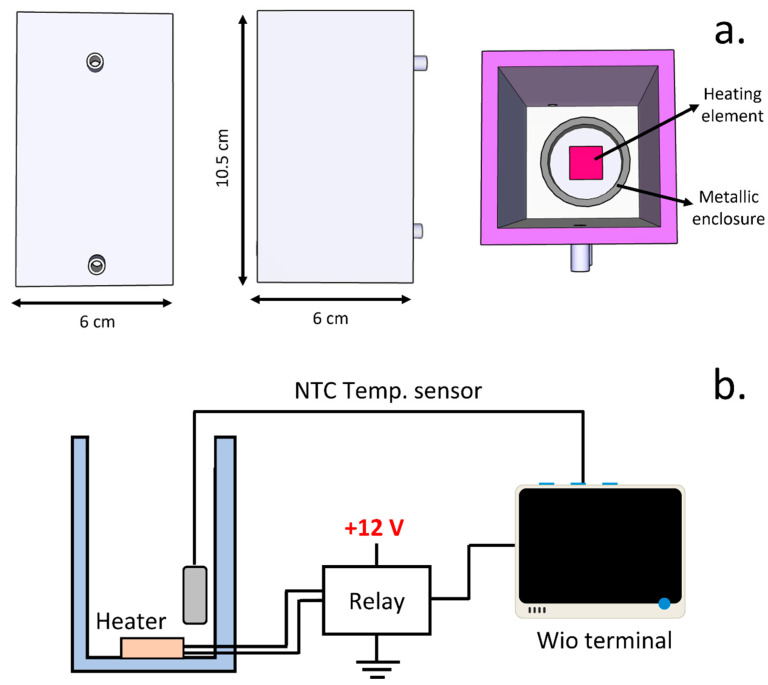
(**a**) Schematical representation of the 3D-printed soil-testing chamber, showing the dimensions and the position of the heating element. (**b**) Circuit diagram of the temperature regulating loop, incorporating an NTC sensor for the measurement of temperature and a heater regulated by a relay. A Wio Terminal device was used for the recording and control of temperature.

To enable the measurement of soil-produced volatiles as well as physical properties (reflectance, impedance, and OCP), a testing chamber was designed. This chamber was fabricated using 3D printing (Ultimaker S3, Ultimaker, Geldermalsen, The Netherlands) with a poly (lactic acid) filament (1.75 mm thickness and printing resolution of 0.2 mm). The testing chamber consisted of a 3D-printed cubic recipient where the soil samples were placed, and a heating element was added at the base to allow a control over the temperature (Figure A1a). An NTC-type temperature sensor was incorporated for the determination of temperature changes in the samples. The information of soil temperature was recorded by a Wio Terminal microcontroller (Seeed Studio, Shenzhen, China), which regulated the heating element using a relay (Figure A1b).

To allow the measurement of soil volatiles, the 3D-printed chamber included two tube fittings where an air pump and a sealed box containing multiple gas sensors were included. The air pump connection was located at the base of the 3D-printed chamber, generating an air flow that circulated through the soil sample and led to the gas sensors. These gas sensors were assembled inside a sealed junction box, where the tube from the 3D-printed chamber as well as the wire connections from the gas sensing devices were inserted through an elastic self-sealing membrane to minimise air leaking. In this work, an air pump was incorporated in the soil chamber to circumnavigate the challenges from non-flow chambers. This pump could avoid the accumulation of gas pressures due to microbial activity, which leads to an inhibition of bacterial growth. Moreover, this method allowed the capture of multiple measurements for each soil sample since sampling could be performed continuously and not within a limited time window to provide data consistency.

The data recorded by the gas sensors were recorded using the Wio Terminal Device. Within this study, metal oxide-based gas sensors including MQ2, MQ3, MQ4, MQ7, and MQ131 as well as BME680 and infrared-based sensors such as SCD30 were incorporated. These sensors allowed a measurement of multiple volatiles and physical parameters (temperature, humidity). The low cost combined with the low limit of detection of these sensors and wide operational range, typically between 0.1–100 ppm, allowed the collection of high-quality data, and it could be used by the widest population.

**Figure A2:**
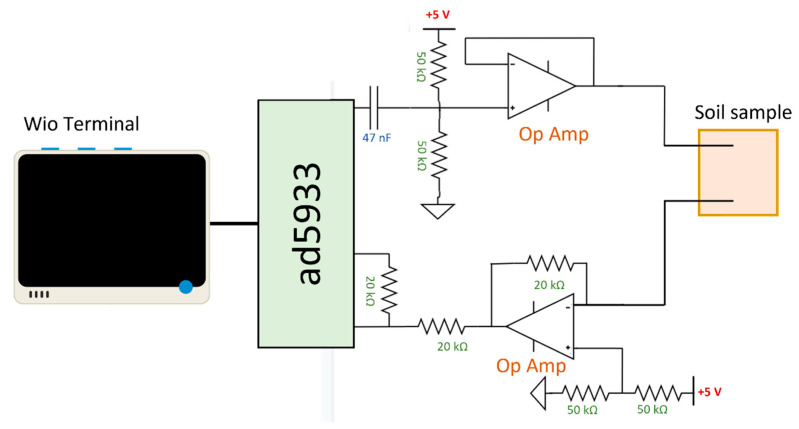
Circuit diagram of the ad5933 device connected to the microcontroller for the determination of impedance spectroscopy. Two electrodes separated by 1 cm were inserted in a soil sample, and the impedance was recorded.

In addition to gas concentrations, physical soil properties were determined. The electrical impedance was measured with an ad5933 device, using the circuit indicated by the manufacturer guidance and detailed in Figure A2. Two electrodes separated by 1 cm were immersed in the soil. The impedance data were then processed using the EIS analyser software (version 1.0) reported by Bondarenko et al. [36]. Both the capacitance and resistance values were estimated using an equivalent circuit consisting of a resistor and a capacitor in parallel, and the LevMarq algorithm was used for the calculation.

To allow a measurement of the reflectance of the soil samples, which provides information about composition, a AS7265x triad spectrometer was also added to the testing chamber. This spectrometer contained a white LED and could record the reflected light intensity from 18 different wavelengths. Finally, a screen-printed electrode was included for the measurement of open-circuit potential (OCP) changes due to bacterial activity. This screen-printed electrode consisted of carbon-based working and counter electrodes, and an Ag/AgCl reference. The differences between OCP from the reference and carbon electrodes were measured using a pH-4502C pH meter, which was connected to the Wio Terminal microcontroller. After incorporating all these physical sensors into the testing chamber, the top side, where the spectrometer as well as the NTC, ad5933 impedance analyser, and OCP connections were installed, was sealed using a flat methacrylate sheet.

### 2.3. Classification of Soil Samples

The possibility of employing the sensing device in the classification of different soil types was explored by using the information provided by the physical sensors (impedance analyser and spectrometer). Both the capacitance and resistance of soil were determined using the ad5933, and reflectance was quantified using the AS7265x spectrometer. This device could measure reflected light within 18 different wavelengths from 410 to 960 nm. Sensors were incorporated inside the soil sensing chamber, and at least three measurements on different samples were taken on each case. All these measurements were determined in soil samples at 25 °C. A principal component analysis (PCA) was finally conducted to determine the capability of our low-cost sensing device in the classification of soil samples. This statistical analysis allowed a reduction in the dimensionality of data, minimising information loss. This method was previously used in the study of electronic noses performance [37]. The first three principal components, containing the highest percentage of variability, were plotted, and the obtained graph was compared to the one obtained after incorporating the impedance measurements.

Spectral emissions from six different soils were determined, either from commercial sources (PureGrow^®^ Peat Free Potting Compost, and Miracle-Gro Premium All Purpose Compost), coir peat, or natural soils from U.K. (lime-rich loamy soils and slightly acid loamy and clayey soils from the North and East of England) and Spain (calcareous soils from the South of Spain).

For the objective determination of the changes on these physical parameters due to changes in soil composition, multiple coir peat/compost compositions were produced by mixing both substrates using different weight ratios. Similar to the previous case, the temperature of each soil sample was first set to 25 °C, and the experiments were repeated in triplicates. Finally, the Pearson correlation coefficients were calculated for each variable using XLSTAT 2021.1, which could be used to determine the most relevant parameters in the study of soil compositions.

### 2.4. Physical–Chemical Analysis of Soil Samples

After the measurement of soil properties using microcontroller-based devices, the morphology of soil particles was studied using scanning electron microscopy (SEM, EVO LS15, ZEISS, Oberkochen, Germany) using an acceleration voltage of 20 kV. In addition, the chemical composition of soil samples was studied by energy dispersive X-ray spectroscopy (EDS, Aztec, Oxford instruments, Oxford, UK) and Fourier-transform infrared spectroscopy (FTIR, L160000A Perkin Elmer, Waltham, MA, USA). These methods offered a good understanding of the concentration of different elements, as well as functional groups in the studied samples. However, they could not be used for the study of the presence of crystalline particles, which is crucial for the study of natural soil samples. In this case, X-ray diffraction (XRD, Philips/PANalytical X’Pert PRO, Panalytical, Malvern, UK) was conducted between 10–60°.

### 2.5. Determination of Soil Volatiles and Seed Germination Rates

The seed germination rates of tomato seedlings after 2 weeks were used as an indicator of soil health. This parameter could be correlated with the concentration of produced volatiles and OCP recorded by sensors inside the testing chamber. In each experiment, 20 g of soil samples were placed inside the testing chamber, and they were incubated for 2 h until a stable temperature was reached. The air pump as well as the gas sensors were then initialised to allow the extraction of gases from soil samples and subsequent measurement. Concentration of volatiles and OCP were quantified each second for 2 h, and the average measurement within the last 1000 data points, once the signal was stabilised, was used for the analysis. Finally, for each soil type, the seed germination rate was determined by planting 10 seeds of the tomato plant, 1 cm deep, and watering them with 5 mL every two days. Seed germination rates were finally calculated.

### 2.6. Synthesis of Molecularly Imprinted Sensors

To allow an objective study of soil mycorrhizal populations, a molecularly imprinted sensor was developed using silica particles. Silica particles were synthetised by condensation of two different siloxane functional groups with tetraethyl orthosilicate (TEOS) binders in the presence of tryptophol templates. β-Cyclodextrin was used as a specific functional group due to its ability to strongly bind indole molecules.

**Figure A3:**
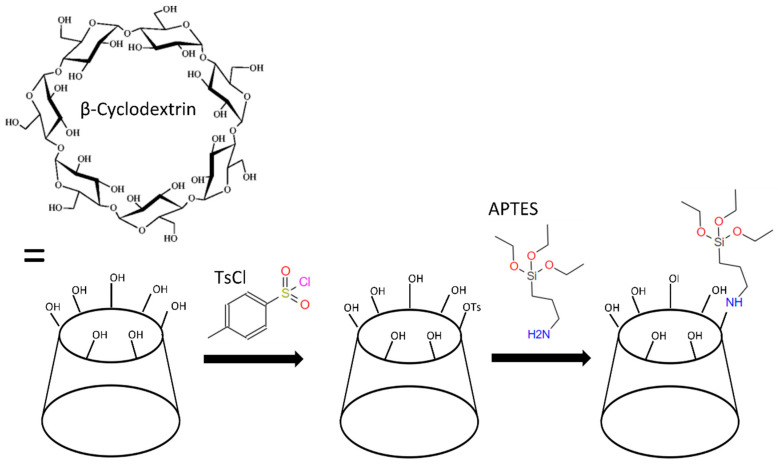
Schematical representation of the chemical synthesis of silyl ether-functionalised cyclodextrin.

A process adapted from Hsieh et al. [38] was implemented to covalently bind β-cyclodextrin to siloxane molecules. This binding was crucial to ensure the formation of specific pores at the silica particle surfaces. Briefly, 220 mL of a 2:1 mixture of β-cyclodextrin and p-toluenesulphonyl chloride was stirred for 2 h. A 40 mL solution of 2.5 M NaOH was added to this mixture, triggering the covalent bond between β-cyclodextrin molecules and p-toluenesulphonyl. The excess of p-toluenesulphonyl chloride precipitated, and it was removed by centrifugation, and the supernatant was collected. Then, 10 g of ammonium chloride was added to the solution containing β-cyclodextrin bound to p-toluenesulphonyl, and the temperature was decreased to 4 °C. A white precipitate appeared, and 1.5 mmol of aminopropyl triethoxysilane (APTES) was added after filtration. This final step allowed the synthesis of silyl ether-functionalised β-cyclodextrin, containing both a β-cyclodextrin pore and a siloxane group, which allowed the condensation with silica monomers. A schematical representation of this chemical reaction is shown in Figure A3.

After modification of β-cyclodextrin to generate the silyl ether-functionalised β-cyclodextrin, the molecularly imprinted silica particles were synthetised. This process was carried out by the condensation of a 20 mM solution of TEOS in the presence of 10 mM 3[(trimethoxysilylpropyl] diethylenetriamine, 10 mM silyl ether-functionalised β-cyclodextrin, and 10 mM of tryptophol used as template molecule in a water ethanol 1:1 (*v*/*v*) solution. The mixture was left to incubate overnight at 4 °C, and the resulting silica particles were purified by centrifugating twice at 4000 rpm for 20 min and redispersing them in DI water. The success of the synthesis was determined by FTIR, showing the peaks corresponding to the characteristic stretches from silica, cyclodextrin, and 3[(trimethoxysilylpropyl] diethylenetriamine. In addition, the morphology of the final particles could be studied by SEM. 

### 2.7. Sensor Fabrication

Molecularly imprinted silica particles were deposited onto screen-printed electrodes by drop casting. A volume of 100 µL of dispersed silica particles was dropped onto the screen-printed electrodes, and the device was left to dry overnight. To ensure uniformity of the silica particle layers, the concentration of dispersed particles was optimised by tuning the concentration of precursors during synthesis. Before drop casting, dispersions were also sonicated to prevent particle aggregation, and samples were slowly dried under controlled drying to avoid irregular deposition. An alginate upper layer was then fabricated by using aerosol-assisted chemical deposition as reported elsewhere [39]. Briefly, 1 mL of a 1 wt% solution of sodium alginate was deposited using a pneumatic nebuliser. An elastomer protective film was finally deposited to improve the device endurance against drought. Similarly to the case of alginate, 0.5 mL of a solution containing 1 wt% of poly (dimethylsiloxane) (PDMS) was deposited by AACD. The thickness of the final device was determined by using a stylus profilometer (Dektakxt, Bruker, UK).

### 2.8. Characterisation of Sensing Device Performance

The screen-printed electrodes were initially calibrated in aqueous solution using multiple concentrations of tryptophol. Concentrations ranging from 10^−7.8^ up to 10^−6^ M were employed, and the measured process was carried on by pulse amperometry using an electrochemical station (Autolab PGSTAT128N, Metrohm, UK). In this case, a 0.8 V was applied for 5 s, and the difference between the initial and last-produced current were quantified. This current difference could be correlated with the concentration of tryptophol. The selectivity of the sensing devices was additionally determined by measuring the sensitivity towards two interference molecules: indoxyl sulphate and N-(3-oxododecanoyl)-L-homoserine lactone. In both cases, the sensors were subjected to the same concentrations of interference molecules in DI water as in the case of tryptophol.

After the calibration of sensing devices using a laboratory-standard equipment, a low-cost microcontroller-based device was designed. This system incorporated a 10 kOhm reference resistor and a digital-to-analogue converter that allowed the microcontroller to apply 0.8 V and measure the changes in current. This configuration could be used to calibrate the screen-printed device using pulse amperometry as in the previous case. Multiple concentrations of tryptophol were then used, ranging from 10^−9^ up to 10^−6^ M. The feasibility of using this system for the determination of tryptophol in soil samples was then studied by immersing the sensing device in a 20:80 coir peat/compost sample. This soil sample was previously sterilised by heating up to 200 °C and then washed using DI water. Different volumes of a solution containing 100 nM tryptophol were then added. Finally, the devices were immersed in a sterilised soil sample, and the changes in tryptophol concentrations were recorded for 9 days. After 4 days, 0.1 g of a commercial mycorrhizal promoter was added to 20 g of soil, and the concentration of tryptophol was measured daily. On each case, tryptophol concentrations were determined in triplicates. In addition, OCP as well as produced volatiles were measured.

### 2.9. Plant Growth Prediction Using an Artificial Neural Network

To enable a prediction of the seed germination rates by using the information collected from soil volatilome and OCP, an artificial neural network (ANN) was used. This ANN was developed by following the work from Bieganowski et al. [40], using the neupy library from python. To train the ANN, a Levenberg–Marquardt learning algorithm was implemented. The total amount of data employed for the training and testing came from 75 soil samples and 31 different measured variables on each case. From each sample, 200 individual measurements were selected and used for the training of the AI algorithms. As such, a total of 15,000 individual measurements were used for the training. From this dataset, 80% of the results were used for the learning and 20% for testing, and the root mean square logarithmic error (RMSLE) and R^2^ were used for the evaluation of the error from the network output. Different AI models were used and compared to find the most optimal algorithm for seed germination rate determination. A sigmoid hidden layer containing 50 nodes was used, and all data were normalised by linear scaling. Moreover, random tree forests and gradient boost were compared using the Sklearn library in python (Spyder 5.4.1).

## 3. Results and Discussion

### 3.1. Respiration Chamber Design

A device that allowed the identification of soil types as well as determination of their fertility was developed. While current electronic noses show high performance in terms of sensitivity and detection limits, reaching sub-ppm levels in most cases, selectivity is a major limitation of devices. In the case of MQ-based electronic noses, which are widely reported in the literature [41,42,43], measurements can greatly vary in complex media due to their generally low selectivity, hindering their ability to identify specific target compounds. Consequently, although these devices have a low cost, they cannot be used to provide an accurate assessment of soil alone. To circumnavigate current challenges in electronic noses, especially given the low selectivity of sensors, a combination of gas sensors operating under different modalities (resistive and optic) and physical sensors was incorporated.

**Figure 1:**
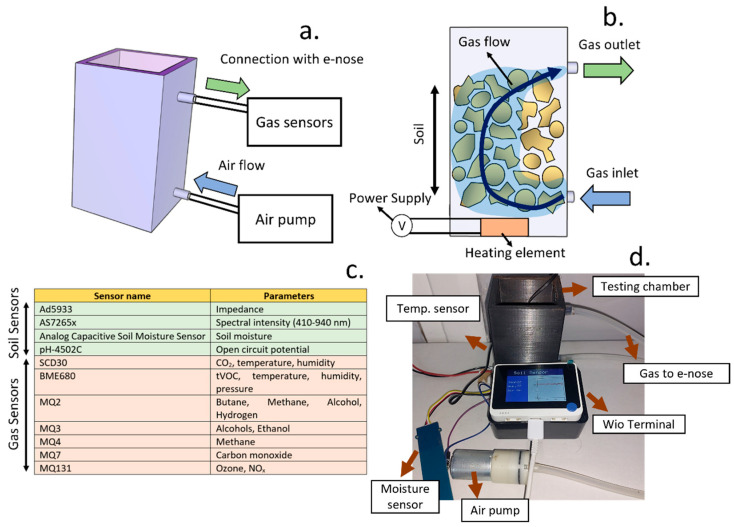
(**a**). Schematic representation of the gas sensing device developed in the present work for the determination of soil parameters and microbial volatilome. The system incorporated two gas connections to generate an air flow. (**b**). The gas flow generated using a pump allowed control of the temperature as well as an active extraction of volatile components generated by microbial communities. (**c**). Complete list of sensors incorporated in the gas sensing device. (**d**). Picture of the final system, incorporating the soil chamber, a thermal control system, as well as an air pump. The results could be visualised using a Wio Terminal device.

This system combined devices for the direct measurement of soil-related parameters that allowed identification (i.e., spectral emissions and electrical impedance) as well as sensors that could be used in the objective quantification of fertility. Sensors were incorporated inside a 3D-printed chamber containing the soil samples (Figure 1a). To allow a standardisation of the soil-testing conditions in terms of temperature, and a heating element was additionally incorporated. This heating element was placed at the base of the soil chamber, increasing the temperature of the circulating gas due to the air pump, and was set at 25 °C. The heated gas flow allowed the system to achieve a homogenisation of temperature in the soil (Figure 1b), which can significantly change the respiration rates and volatile productions from bacteria [44,45], leading to inaccurate results.

The final system incorporated a combination of sensors for the measurement of physical properties from soil as well as the gases generated by microbial communities. A complete list of sensors is included in Figure 1c. This system was also interfaced with a Wio Terminal device, which has an LCD screen to report the results in real time.

### 3.2. Soil Classification Using Low-Cost Sensors

The performance of soil sensors (spectral emission, impedance, and capacitive humidity sensor) was studied in the identification of multiple substrate types. The chosen spectrometer could determine the intensity of up to 18 different wavelengths comprised between the visible, up to near infrared range (410–940 nm). Previous work has shown that soil samples can be classified using hyperspectral imaging techniques. Specifically, the measurement of intensities from near-infrared wavelengths could be used to determine soil nitrogen and organic matter and identify different soil types [13,46,47]. However, in this case, the use of the AS7265x spectral meter device alone could not be used for the accurate identification and classification of soils. Spectral emissions from six different types of soil were determined, either from commercial sources (labelled as peat-free compost 1 and 2, purchased from different brands, and coir peat) or natural soils from the U.K. (labelled as soil 1 and soil 2) and Spain (soil 3).

**Figure 2:**
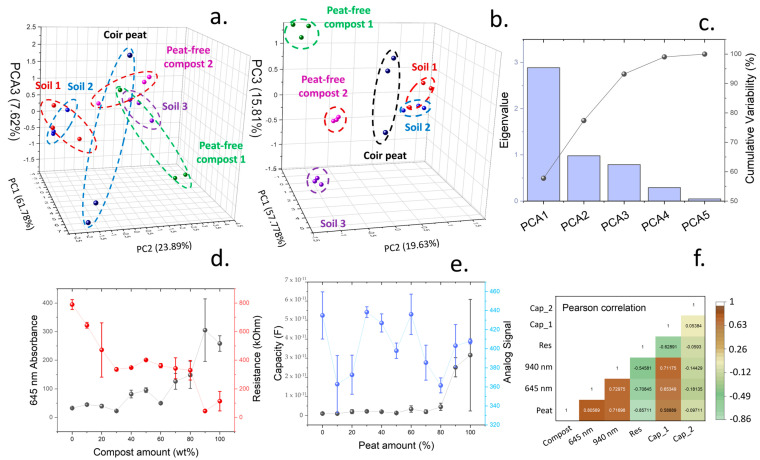
(**a**). Principal component analysis of results obtained after determination of intensity emissions from 18 wavelengths comprised between the visible to near-infrared range. (**b**). Principal component analysis of measurements obtained from the spectral intensities combined with impedance spectroscopy results. (**c**). Contributions to variance of different principal components in shown in (**b**). (**d**). Changes in both resistance, calculated from the impedance plots, and the absorbance at 645 nm in multiple coir peat/compost mixed soils. (**e**). Measured capacitance and moisture (labelled as analogue signal) at different coir peat/compost compositions. (**f**). Pearson correlation for each physical parameter studied in this work and soil compositions.

By using a PCA on the values obtained by the 18 wavelengths, the dataspace could be reduced to a three-dimensional covariance matrix, which retained 93.29% of the sample variance. However, the results obtained after analysis of each soil type led to a poor separation between different samples, hindering their identification (Figure 2a). New soil-specific measurements were then introduced to allow an effective classification. An electrical impedance sensor was incorporated, and the six soil types previously described were characterised. Impedance data were used to calculate both capacitance and charge transfer resistance of soil by fitting using an equivalent circuit consisting of a resistor and capacitor in parallel. Similar models have been developed for soil characterisation to allow a calculation of electrical permittivity [48]. In addition, a soil moisture meter based on a capacitive sensor was incorporated.

After incorporating the new parameters in the PCA, the dataspace was again reduced to a three-dimensions, leading to a small loss of 6.79% of information (Figure 2b). In this case, principal component 1 represented 57.77% of variability, while principal components 2 and 3 represented 19.63 and 15.81%, respectively (Figure 2c). All the samples were grouped in significantly different value ranges, which allowed an easy identification of the different soil types.

**Figure 3:**
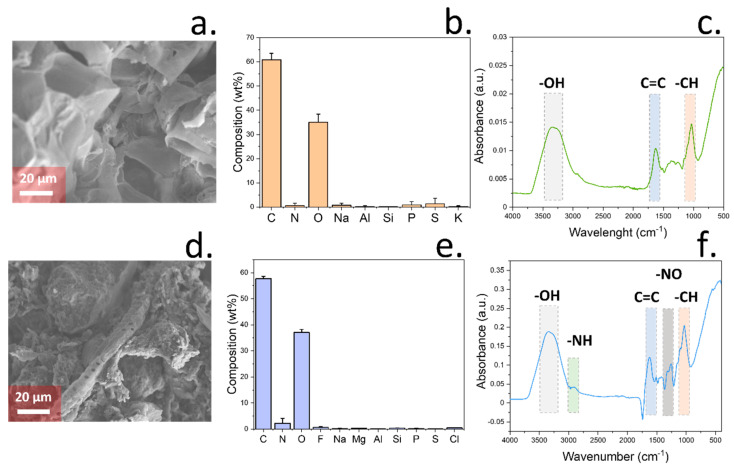
(**a**). SEM imaging of coir peat used for the study of impedance and spectral changes in soil. (**b**). Elemental composition of coir peat samples obtained by EDS. Error bars represent the standard deviation after measuring triplicate measurements. (**c**). FTIR spectrum of coir peat samples. Peaks corresponding to different stretches are indicated. (**d**). SEM imaging of peat-free compost, showing the structural heterogeneity of this substrate. (**e**). EDS compositional analysis of peat-free compost. Error bars represent the standard deviation after measuring triplicate measurements. (**f**). FTIR spectrum of peat-free compost sample. Peaks corresponding to different stretches is indicated. Differences in composition between coir peat and peat-free compost allowed the differentiation. XRD was additionally conducted to further analyse the composition (Figure A4a,b). In the case of coir peat, no diffraction peaks were observed due to the high concentration of amorphous lignocellulose.

Changes in both spectral absorption and soil impedance due to changes in soil composition were further studied by using increasing amounts of coir peat in a peat-free compost. Both soil types presented different compositions, which led to significative changes in their spectral and electrical properties. The incorporation of higher concentrations of coir peat led to a higher intensity of 645 nm, while the charge transfer resistance of the soil decreased. Such an increase of 645 nm reflectance was attributed to a higher concentration of lignocellulosic components in the soil samples (Figure 3d). Similar results were obtained in the measurement of the quantity lignocellulosic materials due to the reflectance of cellulose within the 400–100 nm range [49]. In this case, an increase in lignocellulose also led to a higher concentration of carbon in soil. On the contrary, the measurement of moisture through the capacitive sensor and the electrical capacitance of the soil samples did not show a good correlation with the amount of peat incorporated (Figure 3e). A Pearson correlation matrix containing all the parameters studied is shown in Figure 3f. This matrix evidences the strong relationship between peat concentrations and reflectance at 645 nm, with a coefficient of 0.81, and the charge transfer resistance, showing a coefficient of −0.86.

### 3.3. Chemical Characterisation of Soil Samples

The use of different mixtures of coir peat and peat-free compost allowed the study of changes in soil properties due to increasing amounts of carbon in soil, provided by coir peat. Coir peat is a common substrate with a high content of lignocellulose obtained from coconut husk fibres [50]. This material showed a porous structure when observed by SEM due to the presence of cell walls (Figure 3a). The composition of this material was additionally studied by EDS, revealing a high amount of carbon (60.87 wt%) and oxygen (35.01 wt%) elements, as expected (Figure 3b). The FTIR spectrum of this material presented a high similarity to the spectra reported from lignocellulose materials, showing a wide absorption peak at 3330 cm^−1^ due to the presence of -OH groups in both cellulose and lignin (Figure 3c) [51]. Absorption stretches were additionally observed at 1630 cm^−1^ due to the C = C aromatic vibration [52] as well as the C-H bending vibration within the 1110–1500 cm^−1^ region [51]. Single-bond stretching vibrations were finally found in the 910–1300 cm^−1^ region [53].

Contrary to the morphology observed in the case of coir peat, the compost sample showed a heterogeneous structure, containing plant-derived material and small soil particles (Figure 3d). However, in this case, a higher amount of nitrogen was recorded by EDS, with 2.2 wt% (Figure 3e), along with traces of silicon (0.39 wt%) and electrolytes including Na (0.19 wt%), Mg (0.28 wt%), and Cl (0.46 wt%). When peat-free compost was analysed by FTIR, the obtained spectra presented similarities with the one obtained by coir peat, with peaks at 3300 cm^−1^, 1630 cm^−1^, and 1100 cm^−1^ due to the presence of lignocellulose materials. However, in this case, additional stretches were observed at 2920 cm^−1^ and 1550 cm^−1^, which were attributed to the N-H stretching vibrations and nitrate ions, respectively [54,55]. These results are consistent with the previously observed composition of the compost sample given by EDS, with a higher concentration of nitrogen elements compared to coir peat.

**Figure A5:**
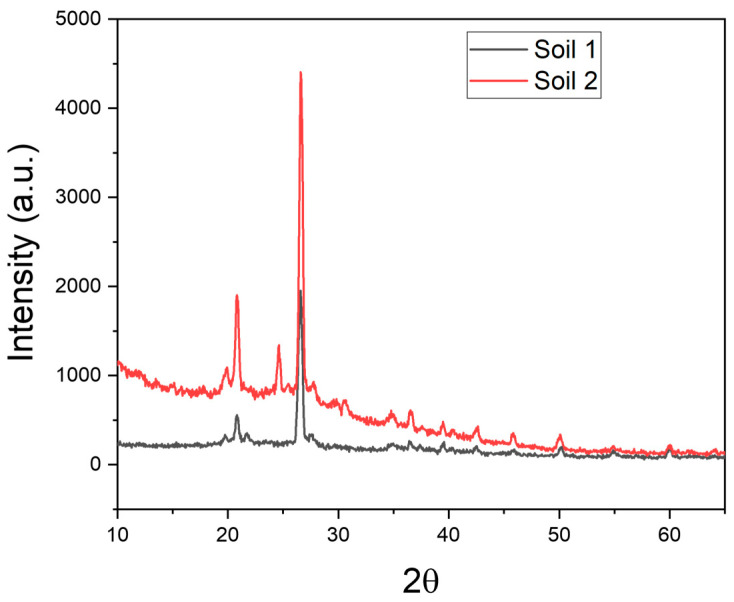
XRD plots of natural soil samples extracted from the U.K. The obtained peaks are consistent with the presence of quartz crystals in soil.

In the case of natural soils, the compositions were similar among the U.K. samples, both containing high amount of quartz crystals, as evidenced by XRD (Figure A5). In addition, the amounts of carbon, oxygen, and silicon in both samples were 41.6 wt%, 19.9 wt%, and 6.7 wt% for soil 1 and 43.8, wt%, 33.6 wt%, and 5.1 wt% in soil 3. The similarities in both crystalline and elemental compositions led to a similar impedance and reflectance values. These results are consistent with the clayey composition reported by the U.K. Soilscapes map, which reports regional differences in soil [37]. These results explain the similarity in the results obtained by principal component analysis.

The data from XRD and EDS could be used for the classification of soil types with high accuracy and the study of nutrients. However, the equipment required is bulky, complicated, time-consuming, and expensive, hindering its use for routine analysis of soil samples. In addition, the information from soil composition alone could not be used directly for the analysis of soil fertility due to the high involvement of soil bacteria in plant growth. To enable a study of soil fertility by the device presented in this work, additional gas sensors were incorporated.

### 3.4. Analysis of Bacterial Activity Through Soil Volatilome and Production of Redox Components

The fertility of soil substrates is dominated by multiple factors, including the concentration of macronutrients and electrolytes [56] and the presence of bacterial [57] and fungi colonies [58]. While the concentrations of macronutrients can be analysed through the chemical composition of soil, the biotic properties of soil cannot be assessed directly in most cases. Within recent years, gas sensors [59] as well as the open-circuit potential of carbon electrodes [19,60] have been employed for the study of these biotic activities. A combination of both approaches was incorporated in the present work to allow a complete analysis of soil health. The seed germination rate from tomato seeds was used as an indicator of soil fertility. This parameter has been extensively studied, as it is influenced by soil salinity [61], acidity [62], and bacterial quality, among others [63]. Within recent years, the impact of soil quality on seed germination rates has been studied, showing a higher impact than moisture [64]. This parameter could be indicative of other soil properties, including nutrient availability and pH [65], soil structure [66], and quality of soil microbiome [67]. As such, the seed germination rates obtained from the different soils could be used for the complete and objective evaluation of soil quality.

**Figure 4:**
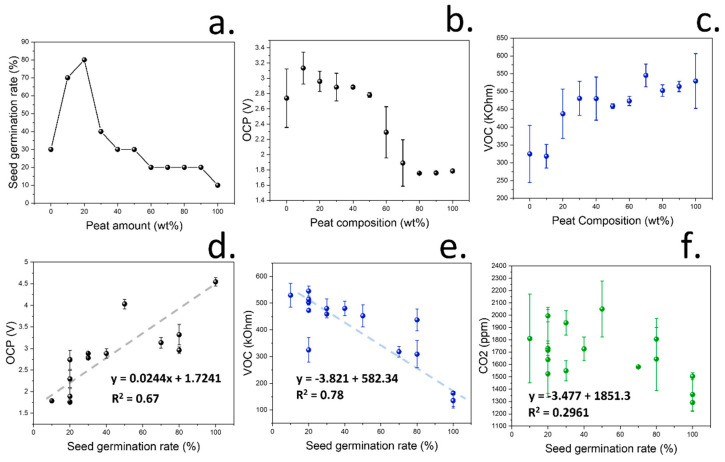
(**a**). Seed germination rates from tomato seeds measured at each combination of soil/compost. (**b**). Recorded changes in OCP at different coir peat/compost compositions. (**c**). Measured resistance values by the VOC sensing device, which are inversely proportional to the concentration of VOCs. (**d**). Determination of open-circuit potential from multiple soil samples and correlation with seed germination rate. (**e**). VOC production from multiple soil samples and correlation with seed germination rates. (**f**). CO_2_ released by multiple soil samples against the seed germination rates. In all cases, the error bars represent the standard deviation after measuring in triplicates.

**Figure A6:**
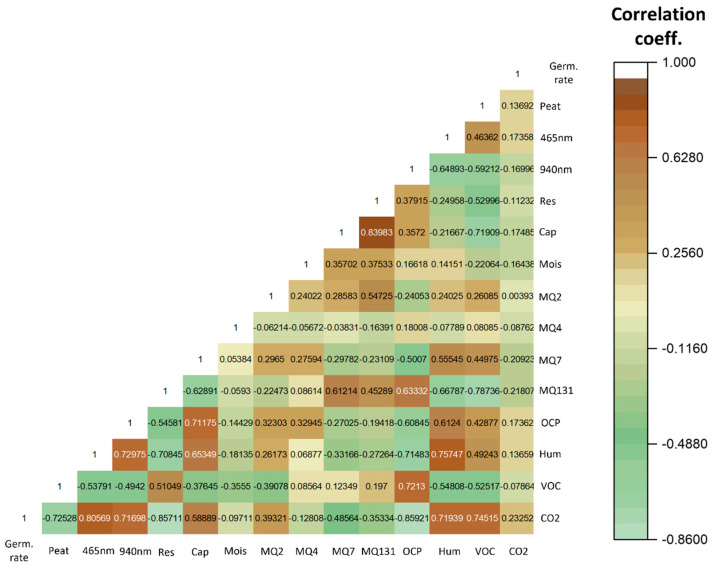
Table showing the correlation coefficients obtained for each variable tested within this work during the study of the effect of peat/compost compositions in the generation of volatiles and seed germination rates.

To study the applicability of the soil-testing device in the determination of fertility, seven different gas sensors were incorporated, which could be used to determine eight gas-related parameters: air humidity, tVOC, CO_2_, reducing gases including H_2_, methane, alcohol, carbon monoxide, and O3/NOx. In the case of tVOC, the electrical resistance of the gas sensor was used for the calculations since it is inversely proportional to the concentration of gas volatiles. The volatile release and OCP changes from the coir peat/compost mixtures developed in the previous section for soil classification were tested, and the seed germination rate was determined. An optimal seed germination rate was measured when a composition of 20 wt% coir peat and 80 wt% of peat-free compost was used, with a germination rate of 80 wt% (Figure 4a). In addition, the higher amount of peat was reflected in a lower detection of OCP and decrease in VOCs (Figure 4b,c), which was expected given the lack of macronutrients and bacteria population in coir peat. A complete Pearson correlation matrix for this experiment, including correlations from all variables, is shown in Figure A6.

After the study of changes in soil properties due to the increase in coir peat, the seed germination rates obtained from the different peat/compost-based soils and natural soil samples were analysed and correlated with the release of organic volatiles and OCP. In the case of the MQ7, used for the analysis of carbon monoxide, no response was obtained by the sensors in any of the experiments. On the contrary, some parameters, such as tVOC and open-circuit potential, showed a good correlation with seed germination rates (R^2^ = 0.78 and 0.67, respectively). This correlation was attributed to the observed relationship of both parameters with the activity of soil bacterial communities (Figure 4d,e). Humidity also showed a strong correlation with seed germination rates and compost amounts due to the higher water retention of peat and the need for a moist environment for seed germination. However, CO_2_ concentrations did not show a direct relationship with seed germination rates (Figure 4f).

As observed, not only the soil properties but also the bacterial activity (reflected in OCP and VOC production) influenced the seed germination rate. These results are consistent with the previously reported work showing high germination rates in the presence of growth-promoting bacteria [67,68]. However, CO_2_ measurements did not show a strong correlation with seed germination rate. CO_2_ production by soil has traditionally been reported to be related with bacterial activity, given its role in the decomposition of soil organic matter [69], and it has been used as an indicator of soil quality, given its effects on plant growth [70]. However, this CO_2_ is normally measured after priming soil with glucose to enhance bacterial metabolism, and it could be influenced by the composition of soil microbiome and not only the quality of soil. Our results also indicate that other parameters, such as OCP or tVOC, show a significantly higher correlation with seed germination rates. As such, the data obtained by the gas sensors highlight the need of a platform that can be used for the detection of multiple gases produced by the soil microbiome since soil fertility cannot be assessed by means of CO_2_ release alone.

### 3.5. Incorporation of Low-Cost, Custom-Made Sensors for the Study of Soil Fungi and Bacterial Colonies Interactions

As shown, the study of soil volatilome and OCP can provide a valuable indication of soil fertility, which is reflected in the seed germination rate. However, these sensors cannot provide accurate information about the composition of these microbial populations. The presence of certain fungal colonies in soil, especially mycorrhizal fungi, has been shown to be beneficial for plant growth and production yields [71,72,73]. However, no fast and reliable methods have been developed so far to enable their measurements in real time. The detection of fungal colonies can be accomplished through the measurement of their quorum-sensing molecules. Compounds such as tryptophol regulate the growth of fungal these colonies [74,75]. As such, the measurement of tryptophol could be used for their indirect quantification in soil.

**Figure 5:**
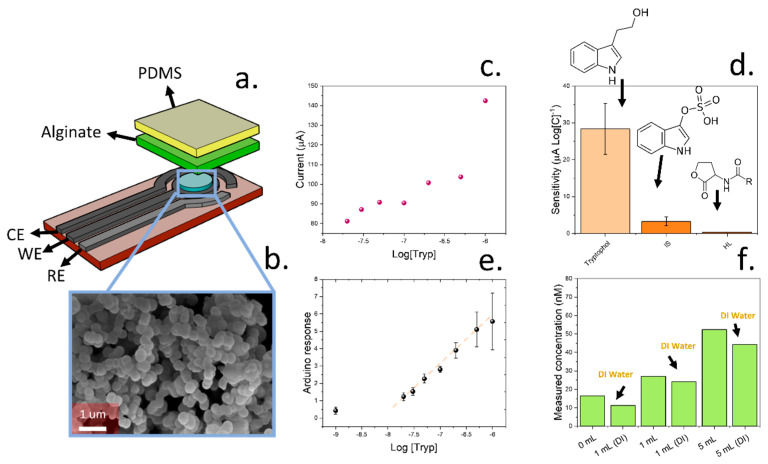
(**a**). Schematic representation of the sensing device based on a three-electrode cell configuration, including the reference, counter, and working electrodes. (**b**). The morphology of molecularly imprinted silica particles was imaged by SEM. (**c**). Calibration plot of screen-printed electrodes in the presence of multiple concentrations of tryptophol. (**d**). Comparison of sensitivities obtained by the sensing devices in the presence of multiple analyte interferences (indoxyl sulphate and homoserine lactone). (**e**). Response obtained by the low-cost Arduino-based device upon subjecting the sensor to multiple tryptophol concentrations. (**f**). Measured tryptophol concentrations in soil samples where different amounts of tryptophol solution had been added.

**Figure A7:**
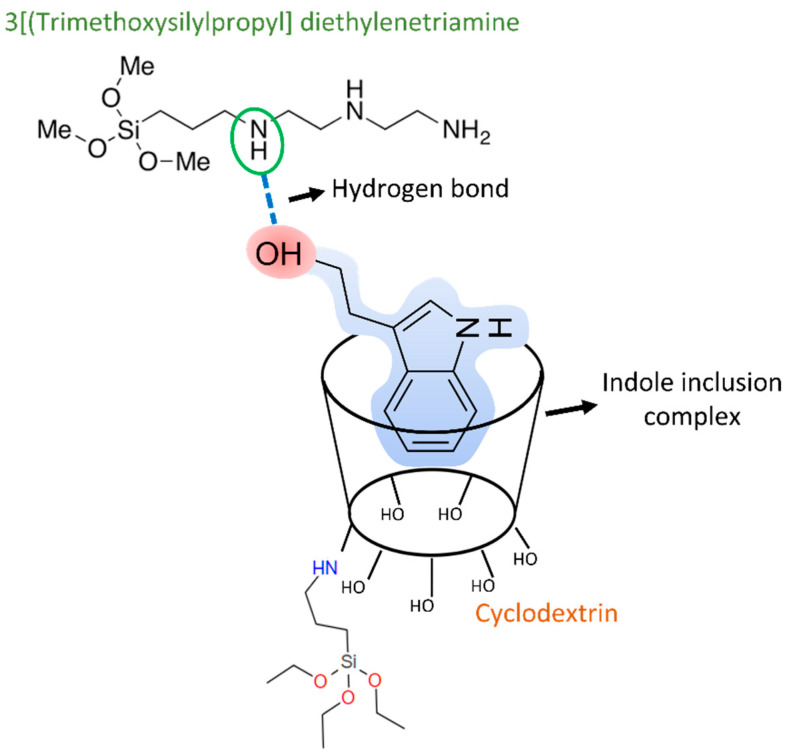
Schematic representation of detection mechanism from the molecularly imprinted silica particles. Such interactions comprise an indole inclusion complex formation by the cyclodextrin molecules and hydrogen bonds due to the aminated 3[(trimethoxysilylpropyl] diethylenetriamine.

**Figure A8:**
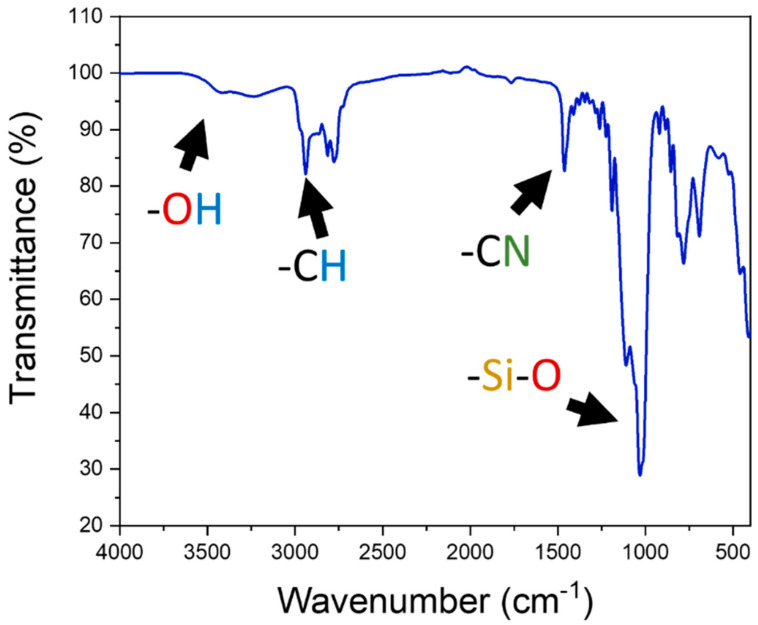
FTIR spectrum of molecularly imprinted silica particles after synthesis incorporating the cyclodextrin and 3[(trimethoxysilylpropyl] diethylenetriamine monomers for tryptophol sensing.

A screen-printed sensing device was designed for the direct quantification of tryptophol in soil samples. The sensor incorporated a three-electrode cell with molecularly imprinted silica fine particles. Alginate hydrogel and PDMS were then deposited as the binding matrix (Figure 5a). Functionalised silica-based particles were synthetised using cyclodextrin and 3[(trimethoxysilylpropyl] diethylenetriamine as the specific functional groups to promote the adsorption of tryptophol (Figure 5b). The combination of cyclodextrin and amine groups allowed a specific adsorption of tryptophol, driven by the strong interactions between cyclodextrin and indole groups [76] as well as the formation of hydrogen bonds with 3[(trimethoxysilylpropyl] diethylenetriamine (Figure A7). The chemical composition of the silica fine particles was confirmed by FTIR after synthesis (Figure A8). Peaks located at 3200, 2900, 1456, and 1026 cm^−1^ were measured, corresponding to the -OH, CH, -CN, and Si-O stretches, respectively [39,77]. The final material was deposited onto screen-printed electrodes by drop casting (Figure 5b), and an alginate layer was fabricated by an aerosol method, as previously reported in our previous work, which showed a good batch reproducibility [39]. In addition, a PDMS film was deposited to prevent the evaporation of water from the hydrogel film. The combination of both alginate hydrogels and protective PDMS film allowed the operation under dry environments and led to a low device thickness of 15.8 µm, as measured by a stylus profilometer. This approach was implemented in the development of mechanically resilient hydrogel-based sensors, which can avoid dehydration by incorporating an elastomer top layer [78,79,80].

Sensing devices were calibrated in DI water using a laboratory-standard electrochemical station. Pulse amperometry was adopted for the sensing. A voltage of 0.8 V was applied for 5 s, and the differences between the initial amperometric current could be correlated with the concentration of tryptophol in solution. This method was adapted from our previously reported work on the detection of indole molecules [39]. Given the low concentration of tryptophol in soil samples, typically in the range of 100 nM/kg_soil_, the devices were calibrated using concentrations ranging from 10^−7.8^–10^−6^ M. The limit of detection was 10 nM, which is lower than the required concentration for assessing fungal colonies in soil. The selectivity of the sensing devices was additionally evaluated using similar indole molecules (indoxyl sulphate) and N-(3-oxododecanoyl)-L-homoserine lactone, a quorum-sensing molecule observed in some bacterial colonies that can be found in soil, such as *P. aeruginosa* [81,82]. The response of the sensitivity of the sensing device towards both compounds was 3 ± 1 and −0.38 ± 0.05 µA Log[C]^−1^, respectively, which is significantly lower than the response observed in the case of tryptophol (28 ± 7 µA Log[C]^−1^), demonstrating the high selectivity of the molecularly imprinted nanoparticles towards tryptophol.

**Figure A9:**
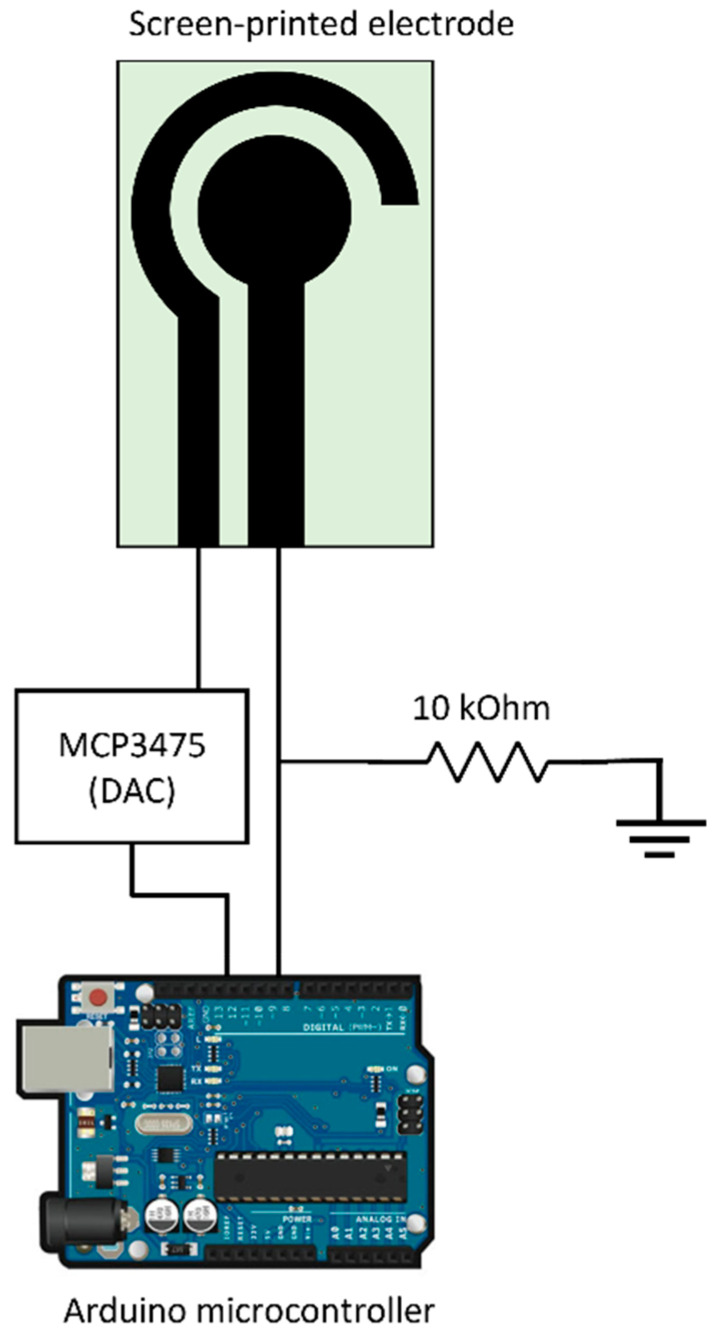
Schematical representation of microcontroller-based circuit employed for the detection of tryptophol, using a reference electrode and a digital-to-analogue converter to apply a 0.8 V.

To enable the use of this sensing device in portable applications, an Arduino-based device was also designed. This device incorporated a digital-to-analogue converter to apply the desired voltage and a current meter using a reference resistance (Figure A9). These sensors were calibrated initially in aqueous solutions using known concentrations of tryptophol, like the previous case (Figure 5e). This calibration revealed a similar limit of detection within the 10 nM range and good linearity below the micromolar level. After calibration in aqueous solutions, the performance of the sensors was tested in soil samples. A 3:7 coir peat/compost mixture was used. This soil sample was sterilised by subjecting it to 200 °C, and possible interferences were removed by two successive washing steps in DI water. The sensing devices were then immersed in soil, and multiple solutions containing 100 nM tryptophol and DI water were used. This experiment allowed the determination of dynamic changes in tryptophol concentrations in soil (Figure 5f).

Measured concentrations of tryptophol in soil were consistent with the magnitude of the volume of tryptophol solution. Upon subjecting 10 g of soil samples to 0, 1, and 5 mL of a solution containing 100 nM tryptophol, the measured concentrations were 16, 27, and 52 nM, respectively. In this experiment, the same volume of DI water was added to the samples immediately after the exposure to different volumes of tryptophol. The measured concentrations were 11, 24, and 44 nM after using 1, 1, and 5 mL DI water, respectively. These experiments evidence the suitability of the molecularly imprinted sensing device for the determination of tryptophol in aqueous and soil samples. This device could be incorporated inside the soil sensing device to allow a complete determination of microbial activity in soil.

### 3.6. Testing of Molecularly Imprinted Tryptophol Sensor Inside Soil-Testing Chamber

**Figure 6:**
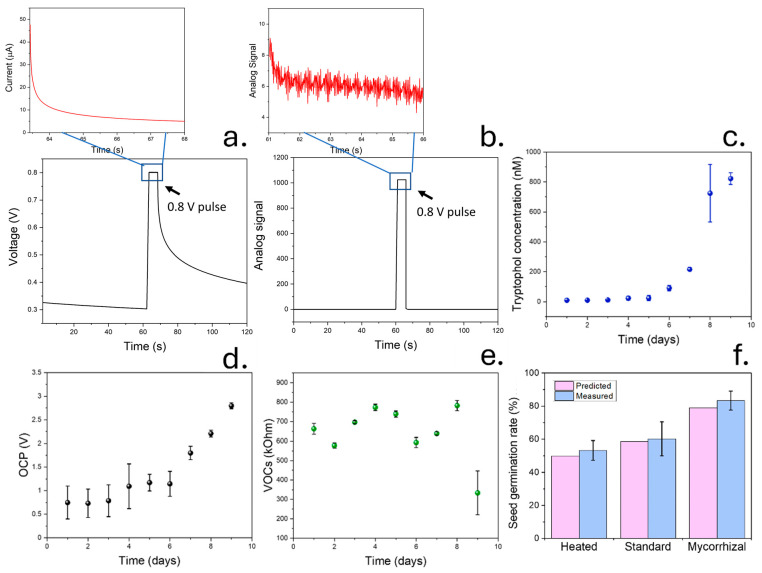
(**a**). Results from pulse voltammetry assay used in the detection of tryptophol molecules in soil. The differences between the initial and last current during the 0.8 V pulse could be correlated with the concentration of tryptophol. (**b**). Pulse voltammetry results obtained after adapting a low-cost Arduino device for the detection of tryptophol. (**c**) The production of tryptophol in soil was evaluated for 9 days using our sensing device. (**d**). All the parameters measured in previous sections related to soil bacterial activity were additionally determined, including OCP. (**e**). Total concentration of VOCs from the soil sample during the 9-day experiment. (**f**). Results comparison between the real and predicted seed germination rates in tomato seeds, using an artificial neural network and three different soil samples.

After the validation of sensing performance of molecularly imprinted silica nanoparticles in soil, devices were tested for 9 days inside a testing chamber. Pulse voltametric signals were recorded and analysed (Figure 6a,b). This experiment allowed the determination of dynamic changes in soil volatiles and biological activity due to the incorporation of mycorrhizal fungi. A 1:4 mixture of coir peat/compost was used in this case since it provided the highest seed germination rate among the tested compositions. Similar to the previous case, the soil samples were initially sterilised by washing using DI water and heating to 200 °C. The produced volatiles as well as OCP measurements and tryptophol concentrations were determined for 9 days, allowing a study of changes in soil microbial activity after subjecting the samples to different conditions. These parameters were initially measured for 2 days in pristine soil after sterilisation, and the samples were then watered. Measured concentrations of tryptophol during these 4 days remained approximately constant, within 10 nM (Figure 6c). This value was similar to the one measured in the previous case using a sterilised pristine soil sample (16 nM). Only after the direct incorporation of mycorrhizal fungi on day 5 was the concentration of tryptophol increased, reaching 822 nM on day 9. A similar concentration was then measured on day 9. Similar results were observed in ectomycorrhizal fungi samples by [83], reporting a concentration of tryptophol in the micromolar range after 10 days of culturing.

The production of volatiles as well as the OCP response of screen-printed electrodes were measured as shown previously (Figure 6d,e). These measurements provided information about the activity of microbial populations in the soil, which are indicative of soil fertility. In the case of tVOC and OCP, which showed the highest correlation with the seed germination rates of plants in previous experiments, the values remained approximately constant within the first 4 days. OCP was in the range of 0.9 V, while the measured resistance from the tVOCs sensors reached 678 kOhm. The low OCP values and high tVOC resistance indicated a small presence of active soil bacteria initially as a consequence of the sterilisation to which the soil samples were subjected before the measurements. The measurements were similar to the ones obtained in the case of pure coir peat that did not contain any living microorganisms. Upon adding mycorrhizal fungi to the soil, the OCP increased, reaching 2.8 V on day 9. This increase in OCP indicates the successful growth of fungal colonies in the soil samples, which had an impact on the seed germination rates of soil.

**Figure A10:**
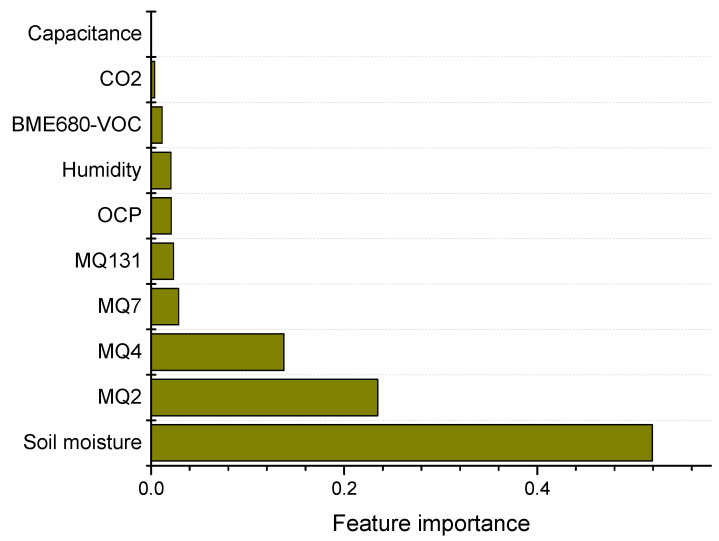
Bar plot showing the importance of the 10 highest variables.

To allow an objective estimation of soil fertility through the measurement of vola-tiles and OCP, different machine learning algorithms were trained, and their performance was evaluated. In total, 15,000 samples from the 75 soil samples were used. An artificial neural network (ANN) was initially trained using a Levenberg–Marquardt learning algorithm. This algorithm can also be designed to not be computationally expensive compared to other methods, which could enable its incorporation into TinyML applications [84], and has been previously incorporated in the characterisation of soil humidity. ANN was additionally compared with other methods, including random tree forest and gradient boost. In all cases, the R^2^ and RSMLE were determined and compared (Appendix A, Table A1). The best performance was observed in the case of gradient boost, with a R^2^ when comparing the calculated and actual germination rates of 0.99 and RSMLE of 0.01. In the case of ANN and gradient boost, while the R^2^ was similar, at 0.97 and 0.99, respectively, the RSMLE was higher (0.1 and 0.03). As such, gradient boost was used in subsequent experiments. The importance of variables was additionally studied. The variables that showed the highest importance are shown in Figure A10.

The gradient boosting algorithm trained using hybrid data could be used for the non-invasive and accurate quantification of seed germination rates after 2 weeks, with similar results compared to the real seed germination rate (Figure 6f). In this case, soil with optimal compost/peat composition that had been heated to 100 °C was compared to standard soil samples and soil with mycorrhizal fungi. The real germination rate was measured after incorporating 10 seeds into each soil type (pristine, watered, and mycorrhizal modified) for 2 weeks.

These results evidence the beneficial impact of soil mycorrhizal populations on seed germination rates. Upon the incorporation of mycorrhizae, both tryptophol concentrations and OCP values increased due to the development of the populations. The trained gradient boost algorithm could be used for the prediction of soil fertility through the measurement of volatiles and OCP. In the case of pristine soil samples after sterilisation, the measured seed germination rate was 53 ± 6%, while the predicted germination rate was 49%. Upon adding the mycorrhizal fungi to the soil samples, both the real and predicted seed germination rates increased to 83 ± 6 and 79%, respectively. These results evidence the ability of gradient boosting to successfully predict seed germination rates.

## 4. Conclusions

In this work, a low-cost soil-testing chamber was developed by the combination of microcontroller-based devices in a 3D-printed case. Multiple gas sensors were used for the profiling of produced volatilome by soil, enabling a measurement of tVOC, CO_2_, and methane emissions, among others. Sensors allowing the direct determination of soil properties including moisture, OCP, impedance, spectral reflectance, and moisture were also included. The determination of these parameters could be used for the classification of different soil types ranging from natural soils to commercially available compost by PCA.

Our system could additionally be employed for the determination of soil fertility through the measurement of produced volatiles and OCP. Seed germination rates of multiple coir peat/compost soils were determined, with a maximum of 80% germination after 2 weeks of measurement. The volatiles produced by 75 different soil samples, including both natural soils and coir peat/compost mixtures, were studied and related to the seed germination rate. A good correlation between microbial activity-related parameters such as tVOCs and OCP and seed germination rates was found (R^2^ = 0.78 and 0.67, respectively).

To allow a complete study of soil biological activity, a miniaturised device based on molecularly imprinted silica deposited onto screen-printed electrodes was also designed. The sensor was operated using a portable circuit, which enabled its direct incorporation into the soil-testing chamber, and was selective towards tryptophol, a common quorum-sensing molecule from fungal populations. The final devices could determine the concentration of tryptophol molecules with high sensitivity and a limit of detection in the range of 10 nM, even when a low-cost Arduino-based circuit was employed. These results show promise for the continuous monitoring of fungal and bacterial populations in soil. A complete study of mycorrhizal populations development was finally conducted using sterilised soil, including the pre- and post-inoculation response of a commercial mycorrhizal promoter. An increase in tryptophol concentration was measured as expected: up to 0.82 µM about 5 days after the use of mycorrhizal promoters. The growth of fungal colonies increased the production of VOCs and OCP, which was reflected by a high seed germination rate.

The full database of soil measurements was used to train a gradient boosting algorithm, which could predict changes in soil fertility in samples subjected to multiple environmental changes. The final algorithm showed good accuracy in the prediction of soil fertility (RMSLE = 0.01), and the results matched the experimental observations. Future work will focus on improving sensor selection to further reduce costs and expanding the database of soil samples to increase the performance of sensors. Moreover, different gas sensors that could provide higher correlations with bacterial activity or seed germination will be incorporated. For example, O_2_ sensors might provide information about seed and bacterial respiration. NH3 sensors, indicative of the presence of ammonifying bacteria, can indicate the abundance of nitrogen nutrients in soil.

## Figures and Tables

**Figure A4:**
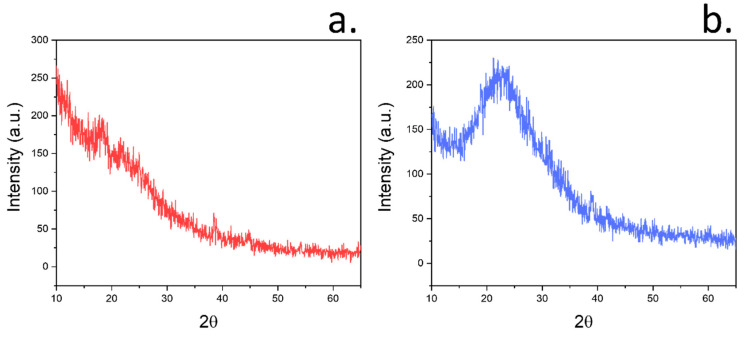
(**a**) XRD plot of coir peat samples, evidencing the amorphous structure. (**b**) XRD plot of compost samples.

## Data Availability

The original contributions presented in the study are included in the article, further inquiries can be directed to the corresponding author.

## Appendix A

**Table A1:** Comparison in the performance between different algorithms used for the prediction of seed germination rates.

	R^2^	RSMLE
ANN	0.98	0.11
Random Tree Forest	0.99	0.03
Gradient Boost	0.99	0.01
